# Risk factors for incident HIV infection among antenatal mothers in rural Eastern Cape, South Africa

**DOI:** 10.3402/gha.v9.29060

**Published:** 2016-01-20

**Authors:** Charles Bitamazire Businge, Benjamin Longo-Mbenza, Verona Mathews

**Affiliations:** 1 Faculty of Health Sciences, Walter Sisulu University, Eastern Cape, South Africa; 2 School of Public Health, University of Western Cape, Cape Town, South Africa

**Keywords:** HIV, incidence, risk factors, antenatal, South Africa

## Abstract

**Background:**

The prevalence of HIV among antenatal clients in South Africa has remained at a very high rate of about 29% despite substantial decline in several sub-Saharan countries. There is a paucity of data on risk factors for incident HIV infection among antenatal mothers and women within the reproductive age bracket in local settings in the Eastern Cape, South Africa.

**Objective:**

To establish the risk factors for incident HIV infection among antenatal clients aged 18–49 years attending public antenatal clinics in rural Eastern Cape, South Africa.

**Design:**

This was an unmatched case–control study carried out in public health antenatal clinics of King Sabata District Municipality between January and March 2014. The cases comprised 100 clients with recent HIV infection; the controls were 200 HIV-negative antenatal clients. Socio-demographic, sexual, and behavioral data were collected using interviewer-administered questionnaires adapted from the standard DHS5 women's questionnaire. Multivariate logistic regression models were used to identify the independent risk factors for HIV infection. A *p*<0.05 was considered statistically significant.

**Results:**

The independent risk factors for incident HIV infection were economic dependence on the partner, having older male partners especially among women aged ≤20 years, and sex under the influence of alcohol.

**Conclusions:**

Therefore, effective prevention of HIV among antenatal mothers in KSDM must target the improvement of the economic status of women, thereby reducing economic dependence on their sexual partners; address the prevalent phenomenon of cross-generation sex among women aged <20 years; and regulate the brewing, marketing, and consumption of alcohol.

## Background

Worldwide, about 2.5 million people become infected with HIV annually, 85% of the infection is through heterosexual transmission (1). South Africa, with about 6.4 million people living with HIV, has the highest number globally and carries about one quarter of the total HIV burden in sub-Saharan Africa (2).

Reducing HIV infections requires rapid and effective scale-up prevention activities (1). It is recommended that countries establish the magnitude of new HIV infections in order to more accurately appraise the success of this scale-up (3).

Substantial decline in HIV prevalence among antenatal clients has been reported in several countries in sub-Saharan Africa (4–6) but not in South Africa despite concerted HIV control strategies (7, 8). Instead, the HIV prevalence among antenatal clients in public health facilities has stabilized at about 29%, and the incidence of HIV among women aged 15–49 years is about 2.28% with about 251,000 new infections per year (2). About 30% new HIV infections in 2007 were in females aged 15–25 years (9).

High HIV prevalence among antenatal clients in public health facilities may be due to persistently high incidence of HIV. The incidence rate of HIV among women in reproductive age in South Africa is more than twice that of the national average for people aged ≥2 years (2). The incidence is highest among females aged 15–24 years, about 2.54% (95% CI: 2.04–3.04) (2).

HIV prevention programs are designed based on incidence and prevalence data collected from national representative samples, which tend to mask the varied risk factors and incidence of HIV in local settings (4, 5). For instance in South Africa, the national antenatal HIV prevalence is about 29%, yet the provincial prevalence is 17% in Northern Cape, 18.4% in Western Cape, 29.3% in Eastern Cape, and 36.7% in Mpumalanga province (7). Therefore, a uniform nation-wide approach toward HIV prevention, without adaptation to the prevailing local social, cultural, and economic contexts, may have contributed to the observed variation in HIV antenatal prevalence across provinces of South Africa.

The aim of this study was to determine the risk factors for HIV infection among antenatal clients attending public health antenatal clinics in King Sabata District Municipality (KSDM) in rural Eastern Cape Province, South Africa.

## Methods

### Study design

This was a case–control study conducted between January and March 2014. Due to the high cost of the Limiting-Antigen Avidity Assay (LAg-Avidity) EIA for identifying clients newly infected with HIV, we used the World Health Organization's criteria for recently acquired HIV infection (newly diagnosed Anti-retroviral drug (ARV)-naïve clients with CD4 counts ≥500/ml and no features of stage 3–4 disease) to define our cases (10). The control group comprised HIV-negative antenatal mothers.

### Study setting

KSDM in OR Tambo District, Eastern Cape Province, is one of the poorest in South Africa with high unemployment, and 88% of the population living below the minimum living level (11, 12). The prevalence of HIV among clients attending antenatal care is about 28.4% (7). The study was conducted at antenatal clinics of two peri-urban PHC clinics and two rural PHC clinics where counseling and testing for HIV is as part of the strategy to prevent maternal to child transmission (PMTCT) of HIV.

**Figure:**
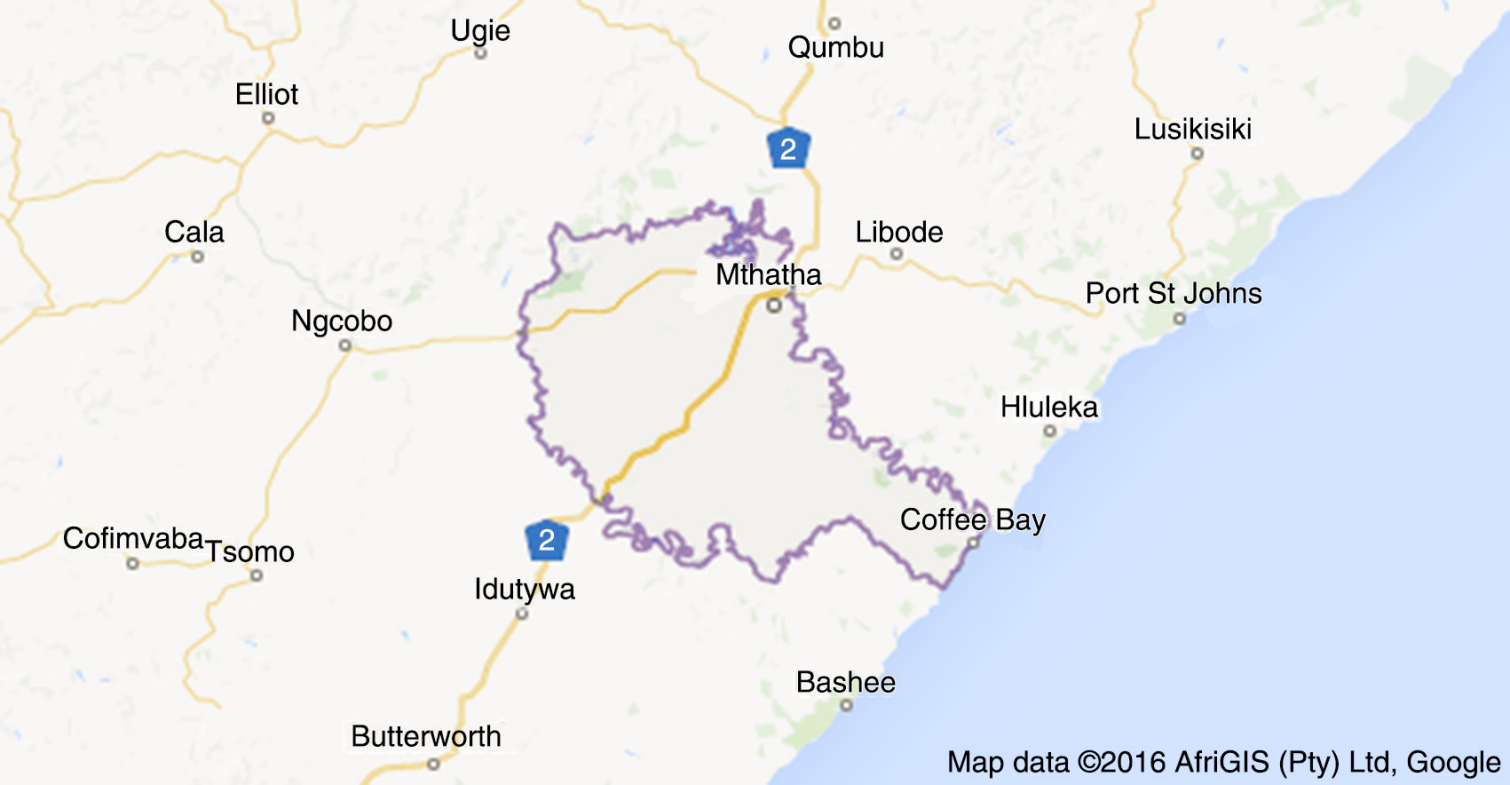
Map showing the geographical location of King Sabata District Municipality, Eastern Cape, South Africa.

### Study population

The study population comprised all clients aged 18–49 years who had never tested HIV positive before the current pregnancy attending antenatal care at public health clinics in KSDM between January 2014 and March 2014.

### Cases

Cases were previously HIV-negative antenatal mothers aged 18–49 years diagnosed with HIV for the first time during the current pregnancy who had CD4 counts ≥500/ml (10). Those with features of HIV stage 3 or 4 were excluded.

### Controls

Controls were antenatal mothers aged 18–49 years who tested HIV negative following counseling and testing for HIV during the current pregnancy.

### Sampling

Mothers who attended antenatal care for the first time at the PHC clinics would undergo counseling and testing for HIV; those who were HIV positive had their blood sample taken for CD4 counts. The mothers who tested HIV positive for the first time, had CD4 counts ≥500/ml and gave informed consent were consecutively enrolled as cases. Following enrolment of a case, the next two HIV-negative mothers who were due for antenatal examination and gave informed consent were enrolled as controls.

### Sample size

In a previous sexual behavior survey among primigravida aged 18–25 years in KSDM, 29% HIV-negative and 61% HIV-positive respondents did not know the HIV status of their regular partners (13). Assuming that the proportion of cases that were not aware of their partners’ HIV serostatus was 0.61, using an online calculator for sample size of unmatched case–control studies (www.stat.ubc.ca/rolling/stats/ssize/htlm), for an odds ratio of 2.5, a desired power of 80% and *α* of 0.05, the sample size for cases was calculated to be 94 which we rounded off to 100 cases. At a ratio of 1 case:2 controls, 200 controls were recruited.

### Data collection

Standardized, structured interviewer-administered questionnaires were used to collect socio-demographic, sexual, and behavioral data for the study. Each interview was conducted in a private room with only the research assistant and respondent in attendance.

### Data processing and analysis

The data were checked for completeness and captured using the Epi-Info statistical package version 3.5.1 and summarized into proportions for categorical variables, means and standard deviation for continuous variables. The Chi-square test was used to delineate the degree of association between categorical variables and HIV infection. The Student's *t*-test was used to compare means between two groups. Univariate odds ratios were computed for association between potential contributing factors and HIV infection with 95% confidence intervals. For avoiding confounding factors and co-linearity, multivariate logistic regression to identify the independent risk factors for HIV infection was performed. Multivariate odds ratios were calculated with 95% CI. A *p*<0.05 was considered statistically significant. All analyses were performed using the IBM SPSS STATISTICS package version 22 for Windows (IBM Inc., Chicago, IL).

### Ethical considerations

Ethical clearance was obtained from the Senate Research Committee of the University of the Western Cape and the Nelson Mandela Academic Hospital Institutional Review Board. Informed written consent was obtained from the study participants before enrolment. The interviews were conducted in places with adequate privacy; we used study codes instead of patients’ names or ID numbers to maintain anonymity. The participants were free to withdraw from the study without fear of retribution.

## Results

### Socio-demographic characteristics

There was no difference in the marital and employment status, and mean number of previous pregnancies of cases and controls. Over 70% of both cases and controls had never married before (Chi-square 0.01, *p*=0.94), with 61% of cases and 70% of controls either not gainfully employed (Chi-square 2.34, *p*=0.13). The mean number of previous pregnancies was 2.1 (SD 1.25) for cases and 2.3 pregnancies (SD 1.18) for controls (*t*=1.32, *p*=0.186). In Table 1, we present the significant univariate association between the respondents’ age, level of education, source of economic support, the main source of HIV-related information, mean age difference with regular partners, and HIV infection.

**Table 1:** Significant association between univariate socio-demographic factor and HIV infection

Variable	HIV negative	HIV positive	Chi-square	*p*
Education level				
Grade 0–Grade 4	1 (0.5)	6 (6.1)		0.010
Grade 5–Grade 8	17 (8.5)	6 (6.1)		
Grade 9–Grade 12	132 (66)	55 (55.6)		
College/university	50 (25)	32 (32.2)	11.46	
Source of economic support				
Parents	87 (43.5)	19 (19)		0.004
Partner	93 (46.5)	65 (65)		
Self	9 (4.5)	7 (7)		
Other	11 (5.5)	9 (9)	17.3174	
Main source of HIV-related information				
Health units only	123 (61.5)	80 (80)		0.034
Health units and school	47 (23.5)	7 (7)		
Health unit/TV/radio + other	27 (13.5)	12 (12)		
Other (magazine/friends/relatives)	3 (1.5)	1 (1)	13.57	
Variable	Mean±SD	Mean±SD	*t*-statistic	*p*
Mean age of respondents	24.6±5.5	26.2±4.9	2.41	0.015
Mean number of sexual partners	2.8±1.7	3.6±1.7	3.47	0.006
Mean age of regular partner	29.2±6.9	32.0±6.5	3.81	0.001
Mean age difference with regular partner (years) Age group of respondents (years)	Mean difference±SD	Mean difference±SD	*t*-Statistic	*p*
<0	3.0±2.3	10.0±4.2	6.55	<0.001
20–24	5.1±4.0	5.1±2.9	0.001	0.992
26–29	5.5±3.6	5.6±4.0	1.770	0.860
30–42	4.5±3.2	6.7±6.1	1.860	0.068

### Bivariate odds ratios for incident HIV infection

The risk of HIV infection was mainly associated with the age, marital status, alcohol use, and economic dependence of the respondent on one hand and the age, number, and employment status of sexual partners on the other hand.

### Independent risk factors for incident HIV infection

Factors with co-linearity (age of respondent and knowledge of HIV status of partner, and related behavior risk factors) were not included in the multivariate logistic regression analysis. After adjusting for confounding factors (educational level, addictive habits, sex without condom under the influence of alcohol, number of partners, employment of partners, concurrent partners, vaginal discharge, and other sexual partners) using logistic regression analysis, and forward Wald strategy, only older age of partner, economic support source, and sex under alcohol were identified as the most important and significant risk factors for HIV acquisition in these pregnant women (Table 2).

**Table 2:** Independent risk factors for HIV infection

Independent variables	B	SE	Chi-square Wald	OR	95% CI	*p*
Main source of economic support						
Partner	2.770	0.406	46.530	16	7.2–35.4	<0.0001
Family/other			Reference	1		
Engagement in sex under the influence of alcohol						
Yes	3.048	0.508	36.004	21.1	7.8–57	<0.0001
No			Reference	1		
Age of partner						
≥25 years	3.096	0.495	39.142	22.1	8.4–58.3	<0.0001
<25 years			Reference	1		
Constant	−5.502	0.671	67.165			<0.0001

## Discussion

### Socio-demographic and behavior characteristics associated with incident HIV

The present study revealed diverse factors associated with higher risk of HIV transmission among poor black women during pregnancy in Eastern Cape Province, South Africa. The univariate contributing factors of HIV infection during pregnancy such as chronological age, socio-economic status, sexual risky behaviors, and lifestyles identified in this study, are well established in the literature (2, 14, 15).

Our findings are consistent with studies which revealed a higher risk of HIV among women aged >25 years (15). The latest South African HIV sero-survey showed that frequency of condom use was greater among youth aged 15–24 years than among older people (2).

HIV prevention programs that are focusing on education among the youth in the media, schools, and churches in South Africa may account for the observed difference (16, 17). Indeed in the current study, the risk of HIV was more than 15-fold higher among respondents with less education (Table 3). However, a lower educational level is also associated with low socio-economic status that may partly account for the observed high risk.

**Table 3:** Bivariate odds ratios of HIV infection

Variable	HIV positive, *N* (%)	HIV negative, *N* (%)	OR	95% CI	*p*
Total	100	200			
Age of respondent					
≥25	52 (41.9)	72 (58.1)	1.93	1.15–3.23	0.012
<25	48 (27.3)	128 (72.7)	1		
Age of regular partner					
≥25	91 (40.1)	136 (59.9)	4.76	2.16–10.82	<0.001
<25	9 (12.3)	64 (87.7)	1		
Education status					
Grade 0–Grade 4	7 (70)	1 (30)	14.98	1.82–328	0.031
≥Grade 5	93 (32.1)	199 (67.9)	1		
Source of economic support					
Partner/self or other	81 (41.8)	114 (58.2)	3.22	1.75–5.95	<0.001
Parents	19 (17.9)	86 (82.1)	1		
Smoking					
Yes	15 (50)	14 (50)	2.34	1.02–5.42	0.027
No	85 (31.6)	186 (68.4)	1		
Dagga/cannabis use					
Yes	7 (63.6)	4 (36.4)	3.69	0.94–15.42	0.065
No	93 (32.2)	196 (67.8)	1		
Alcohol consumption					
Yes	34 (40)	50 (60)	1.55	0.89–2.69	0.161
No	66 (30.7)	150 (69.3)	1		
History of addictive alcohol consumption					
Yes	20 (69)	9 (31)	5.31	2.18–13.22	<0.001
No	80 (29.5)	191 (70.5)			
Number of alcoholic drinks of respondent					
≥6	22 (51.2)	20 (48.8)	2.54	1.25–5.17	0.012
0–5	78 (30.4)	180 (69.6)			
Ever engaged in sex under influence of alcohol					
Yes	18 (63)	10 (37)	4.17	1.73–10.20	<0.001
No	82 (30.4)	190 (69.6)			
Sex without condoms due to alcohol					
Yes	18 (60)	12 (40)	3.44	1.49–8.01	0.002
No	82 (30.4)	188 (69.6)	1		
Number of lifetime partners					
≥2	94 (38.1)	154 (61.9)	4.68	1.83–12.70	<0.001
1	6 (11.3)	46 (88.7)	1		
Employment status of regular partner					
Employed	94 (39.2)	130 (60.8)	8.44	3.35–22.55	<0.001
Scholar/unemployed	6 (19.3)	70 (80.7)	1		
Knowledge of HIV status of partner					
No/not known	44 (47.8)	47 (52.2)	2.56	1.48–4.41	<0.001
Yes (known (±))	56 (27.1)	153 (72.9)	1		
Whether regular partner had other sexual partners					
Yes/not sure	88 (37)	149 (63)	2.51	1.22–5.27	0.014
No (r)	12 (19.4)	51 (80.6)			
History of concurrent partners					
Yes	15 (50)	15 (50)	2.18	0.96–4.96	0.066
No	85 (31.5)	185 (68.5)	1		
Episodes of vaginal discharge in the previous 12 months					
≥1	74 (42.8)	98 (57.2)	2.96	1.70–5.19	<0.001
None (r)	26 (20.50)	102 (79.5)			

“r” indicates the reference group.

Consistent with findings by others, binge alcohol consumption and alcohol addiction increased the risk of HIV infection in the study population especially among respondents aged <25 years (2, 15, 18, 19). This was attributed to multiple sexual partners and inconsistent condom use among high-risk alcohol consumers.

Among rural communities in South Africa, homemade alcohol is an economic activity that also avails alcohol at affordable cost (19, 20) potentially leading to early alcohol initiation and high dependency among youth (12, 21). The regulation and control of alcohol marketing has been shown to delay onset of drinking, binge consumption, alcohol dependence (22), and incidence of sexually transmitted diseases (23).

The increased risk of HIV infection in the current study arising from the use of psychoactive drugs such as dagga/cannabis may be attributed to reduced inhibition for transaction sex, improper condom use and inability to negotiate protected sex in an effort to circumvent sexual and physical violence (24).

In the current study, we observed more than a fourfold increase in the risk of HIV among respondents who had older partners. This underscores the importance of interventions targeting inter-generational sex in the prevention of especially among respondents aged <20 years. Older male partners are perceived as more ready to meet the demands of the young women in school or urban areas than their same-age mates (25).

Consistent with findings from other settings in sub-Saharan Africa (26, 27), majority of HIV-positive respondents in the current study were dependent on their partners for financial support. Economic dependence on sexual partners is associated with reduced capacity to negotiate protected sex and increased risk of casual sex (1, 25, 28). Economic dependence on partners in KSDM may be a result of the rampant poverty given that 75% of households are dependent on social welfare grants and 88% of the population lives below the poverty line (12).

In the current study, we found that the risk of HIV infection among the respondents increased with the number of lifetime sexual partners, consistent with a review of demographic health surveys of 22 sub-Saharan countries, in which the HIV prevalence was higher among respondents who had three or more lifetime partners (29). Concurrent as well as frequent serial sexual partnerships significantly increase the risk of HIV transmission (2, 30).

In our study, having a regular partner of unknown HIV serostatus was associated with increased risk of HIV. Individuals who have established their HIV serostatus tend to take precautions to prevent infection of their partners (31, 32). This is less likely to be the case where a high risk of HIV was observed in cohabiting (2) and married couples (15, 33). Partners in long-term relationships consider condom use undesirable as it equates to mistrust (34), increasing the risk of HIV infection among women with partners of unknown HIV serostatus. The unknown HIV status of sexual partners may imply lack of HIV prevention services for couples and hard to reach populations such as men (35, 36) in KSDM. Women in casual relationships are rarely aware of the HIV status of these partners (37) and are therefore less empowered to demand protected sex.

Although HIV prevention information can be accessed by individuals through the media, community-based interventions and health facilities (16), our data show that the majority of HIV-positive respondents received HIV prevention information from the health units, probably after sero-conversion. Johnson et al. (38) found that 13% of the population in the Eastern Cape South Africa had not had access to even one HIV communication prevention program. Shisana et al. (2) in a recent South African national HIV survey found a reduction in HIV prevention knowledge. They attributed this to a change of focus from social and behavioral approach to mainly biomedical health unit based interventions such as male circumcision and Anti-retroviral therapy (ART) and the prevention of maternal to child transmission of HIV (PMTCT) using ART instead of both approaches.

The findings of the current study suggest that lack of adequate HIV prevention information and limited exposure to HIV prevention programs may be associated with an increased risk of HIV infection among respondents in urban informal residential areas in KSDM. This may be a result of poor infrastructure and lack of programs that specifically target the informal settlements in urban areas (2).

The number of abnormal vaginal discharge episodes in the previous 12 months before the study was associated with increased risk of HIV infection. This is consistent with observations from other researchers (15, 39, 40). This may reflect the associated risky social behavior that predisposes to other sexually transmitted infections (STI) in addition to HIV (39). However, individuals who live among populations with a high prevalence of STI have previously been shown to be prone to increased risk of HIV acquisition (1, 41). This underscores the importance of behavior change for both members of a couple and that of prevention, early and effective treatment of sexually transmitted diseases in the general population as strategies for HIV prevention.

### Independent risk factors for incident HIV infection

Women in reproductive age in rural Eastern Cape, South Africa, are at increased risk of HIV infection due to inter-generational sexual relations. This is consistent with the recent South African HIV sero-survey in which cross-generation sex was an important risk factor of HIV among women aged 15–24 years (2). Sexual relationships with older men are often driven by financial and material gains (25) and characterized by reduced capacity to negotiate safe sex (1, 28). This may explain the observed high-risk of HIV associated with sexual partners aged >25, and economic dependence on sexual partners.

Sex under the influence of alcohol was another independent risk factor for HIV infection among antenatal mothers in KSDM. It is not only associated inconsistent of condom use as observed in the current study, but also with *incorrect* use of condoms even among respondents who reported consistent condom use (2). High-risk alcohol use is a risk factor for multiple sexual partners and other sexually transmitted diseases, all of which are known risk factors for HIV infection (15). The increased risk of HIV due to alcohol in rural Eastern Cape could be as a result of the high prevalence of homemade alcohol, which is a well-recognized economic activity among women in rural areas in South Africa (19, 20). This highlights the potential role of exploring interventions that impart alternative economic skills for women in rural Eastern Cape, South Africa, and other multisectoral interventions against HIV.

### Recommendations

Poverty alleviation programs in KSDM should include specific efforts to improve the welfare of women so as to reduce dependence on sexual partners or on home-based alcohol production.

There is a need to explore the access, attitude, and acceptability of the current HIV interventions and their impact on behavior change.

Further research is required to identify factors that make alcohol and psychoactive substances available in both rural and urban areas of KSDM and the marketing forces that endear them to the youth.

The impact of current alcohol advertisements on the initiation of alcohol consumption and dependence needs further study.

It is necessary to ascertain ways through which the local government can work with the communities and schools to identify local alcohol production and regulate all alcohol outlets within the community, including the identification and treatment of individuals who are addicted to drugs and alcohol.

### Study limitations

Recall errors, underreporting, and social desirability may have affected the quality of our data (42). Our method of determining recent HIV infection compared to newer but more expensive Limiting-Antigen Avidity Assay (LAg-Avidity) EIA has more potential of misclassifying respondents with chronic HIV as having incident HIV infection.

The association between different variables and an HIV sero-positive status cannot prove causality.

While the interviewer-administered questionnaire ensured completeness of the data, a potential observer bias may have been introduced, as the research assistants were not blinded as to whether the participant was a case or control when they were administering the questionnaires.

## Conclusions

Our study reveals that the main risk factors for HIV in KSDM are economic dependence on sexual partners, risky alcohol consumption and associated inconsistent condom use, and cross-generational sex especially among women aged ≤20 years. Therefore, effective prevention of HIV among antenatal mothers in KSDM must target the improvement of the economic status of women, thereby reducing economic dependence on their sexual partners; address the prevalent phenomenon of cross-generation sex among women aged ≤20 years; and regulate the brewing, marketing, and consumption of alcohol.

## Authors' contributions

Charles Bitamazire Businge (CBB) and Verona Mathews (VM) designed the research project. CBB carried out data collection. CBB and Benjamin Longo Mbenza (BLM) did the data analysis. All the three authors participated in writing the manuscript.

## Conflict of interest and funding

There is no conflict of interest. CBB is a recipient of a pilot research grant (CFAR grant P30 AI036214) from the Centre for AIDS Research at the University of California San Diego (CFAR UCSD), USA. The authors have not received any other funding or benefits from industry or elsewhere to conduct this study.
